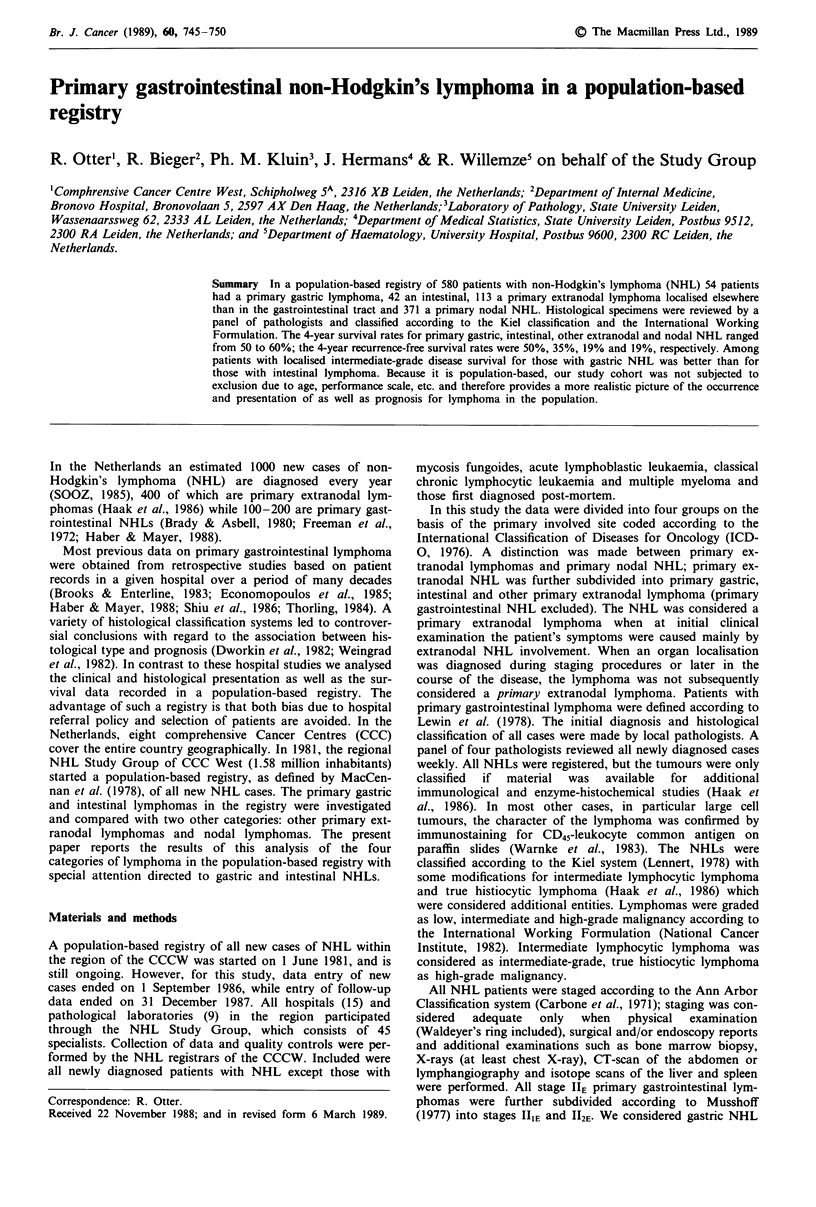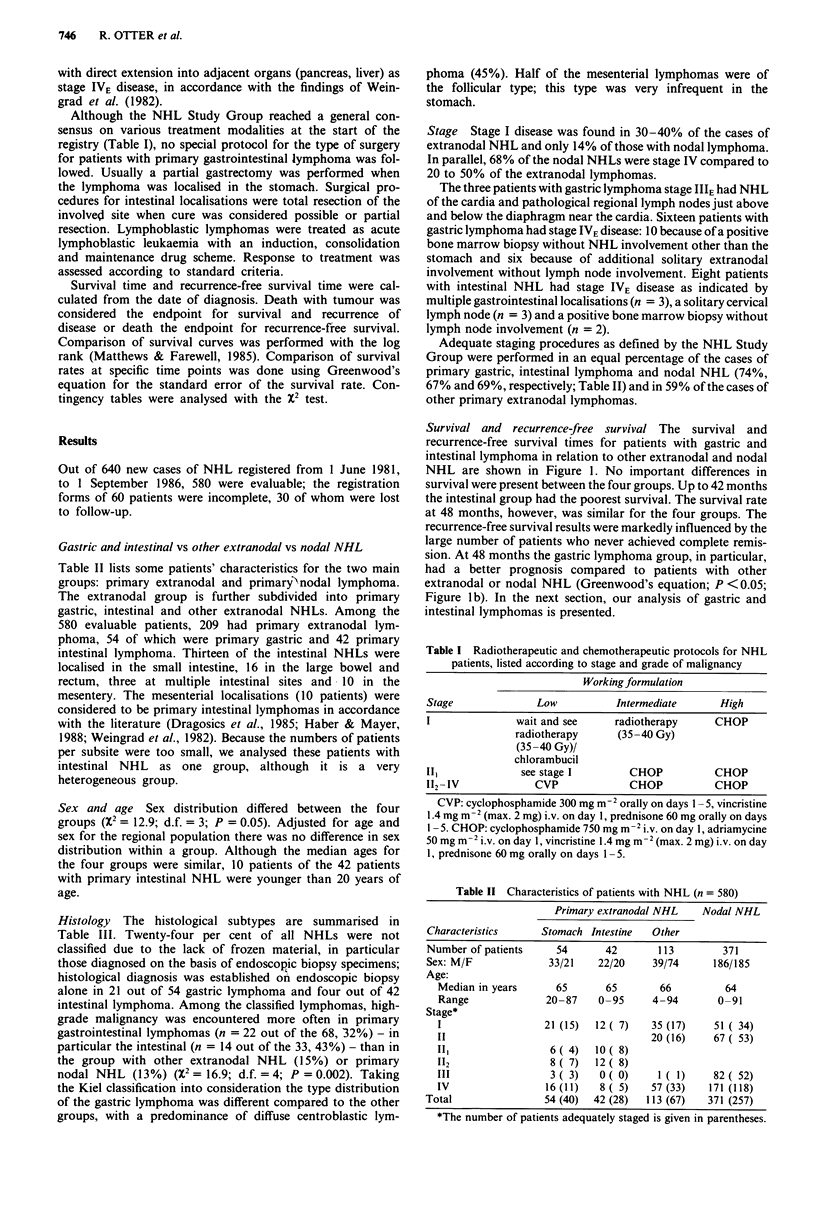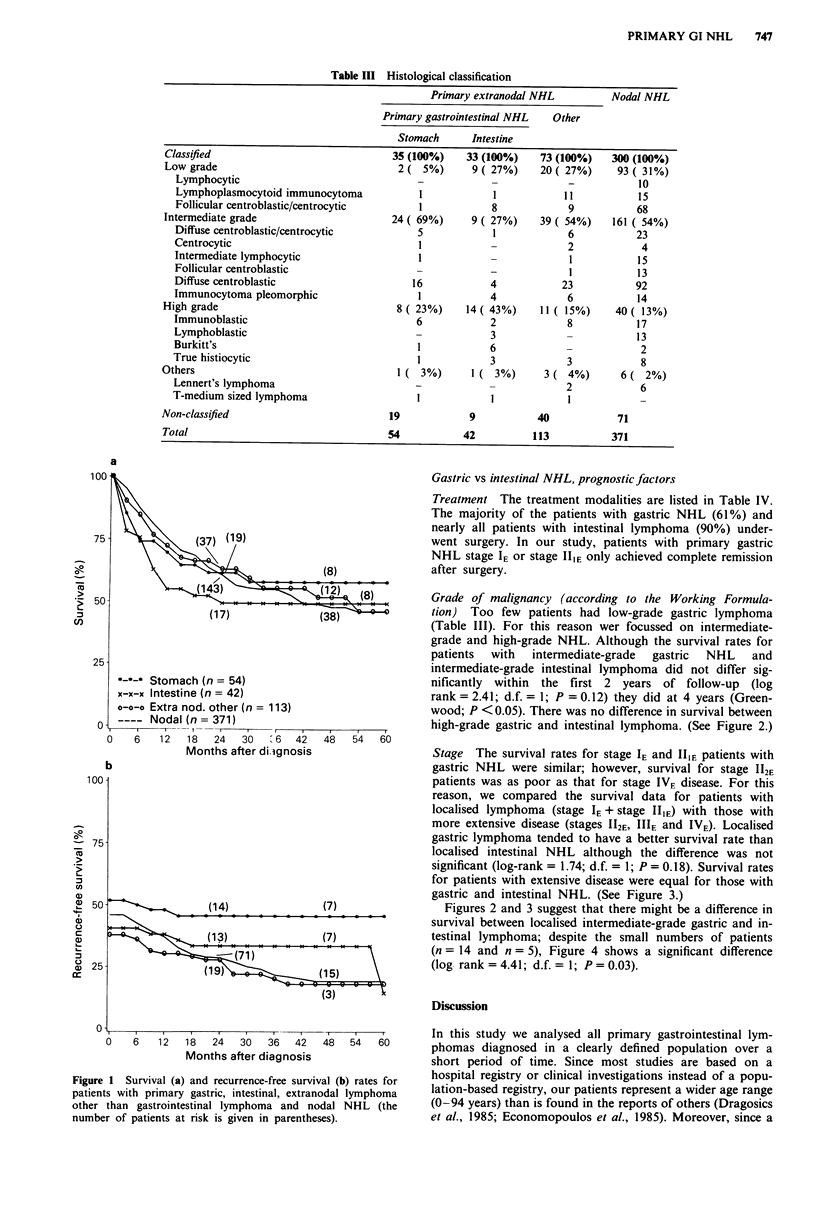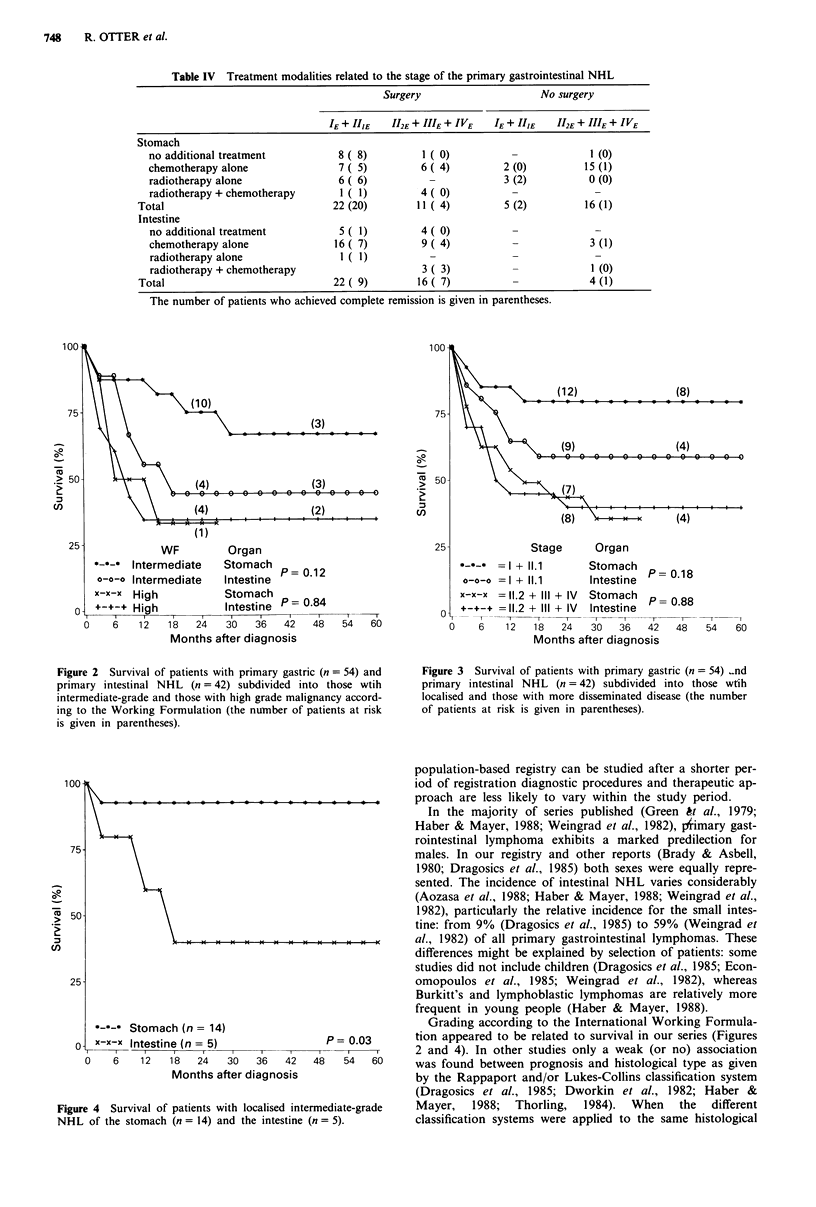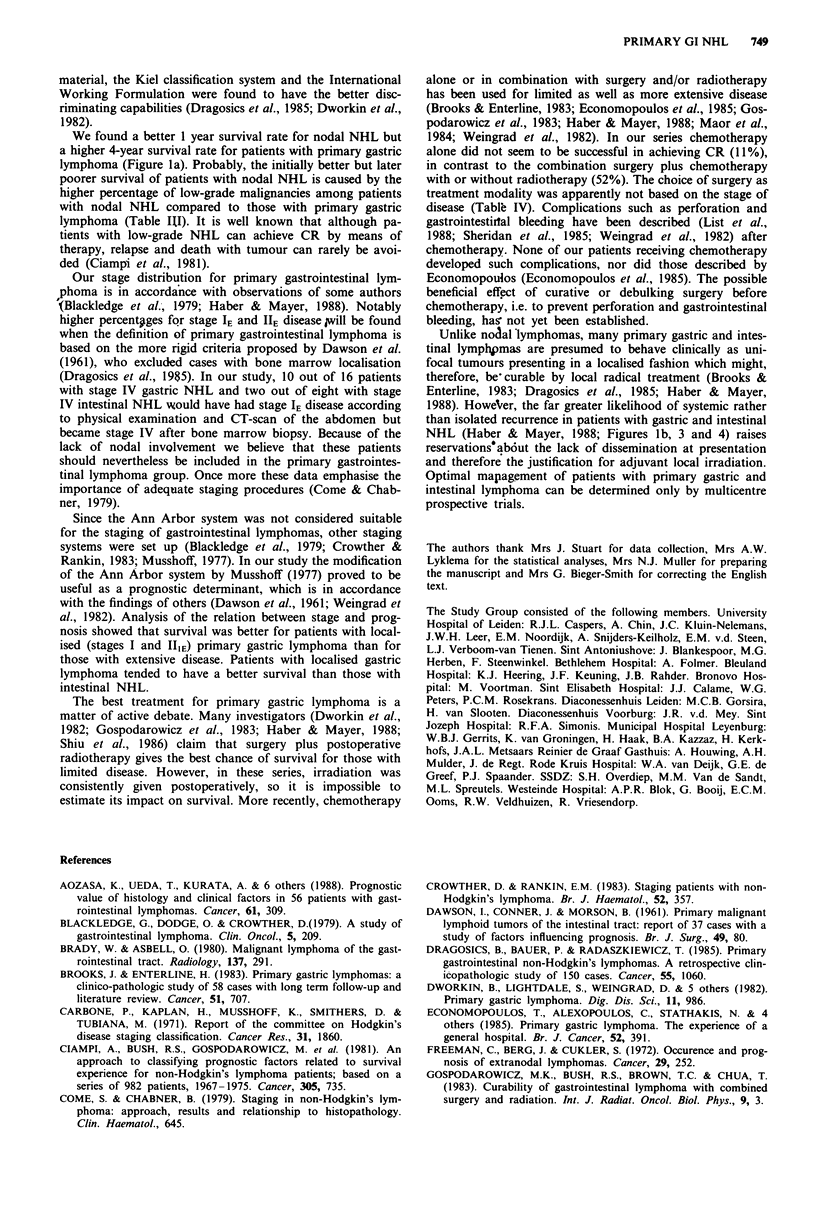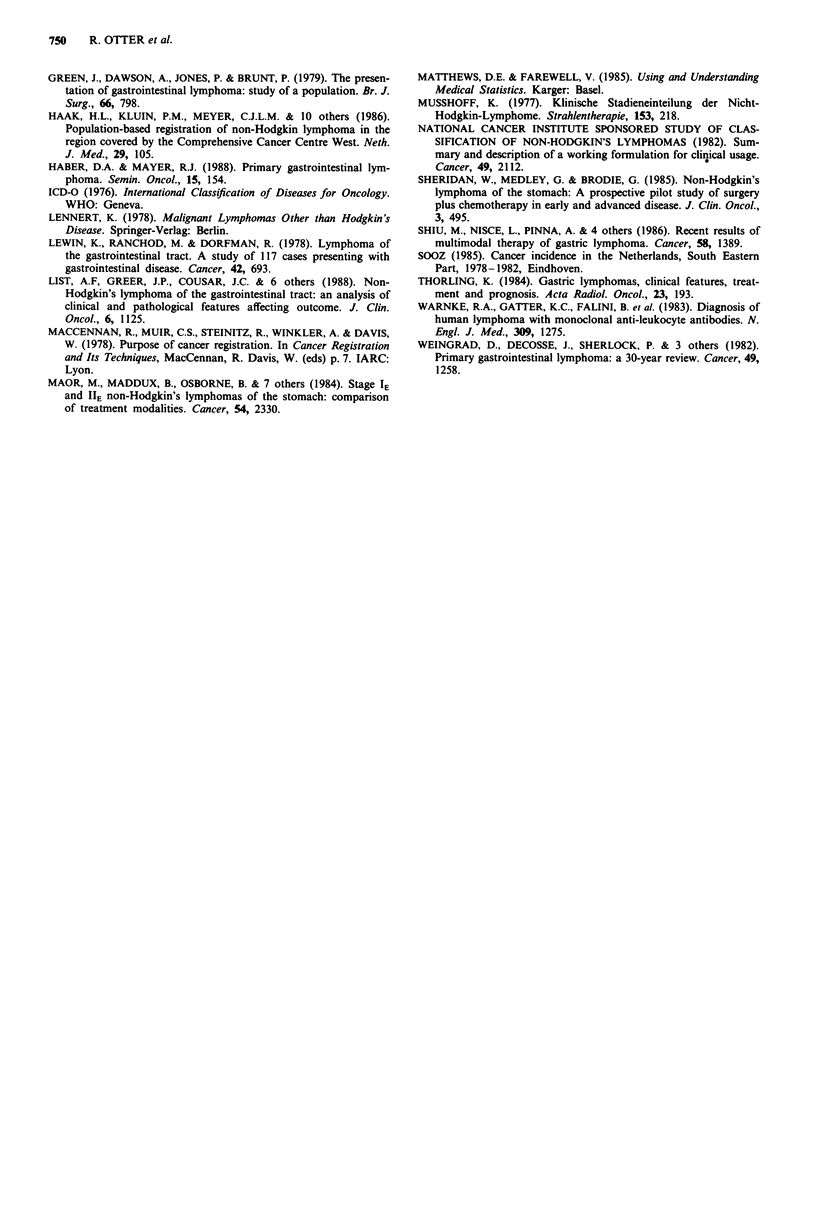# Primary gastrointestinal non-Hodgkin's lymphoma in a population-based registry.

**DOI:** 10.1038/bjc.1989.351

**Published:** 1989-11

**Authors:** R. Otter, R. Bieger, P. M. Kluin, J. Hermans, R. Willemze

**Affiliations:** Comphrensive Cancer Centre West, Leiden, The Netherlands.

## Abstract

In a population-based registry of 580 patients with non-Hodgkin's lymphoma (NHL) 54 patients had a primary gastric lymphoma, 42 an intestinal, 113 a primary extranodal lymphoma localised elsewhere than in the gastrointestinal tract and 371 a primary nodal NHL. Histological specimens were reviewed by a panel of pathologists and classified according to the Kiel classification and the International Working Formulation. The 4-year survival rates for primary gastric, intestinal, other extranodal and nodal NHL ranged from 50 to 60%; the 4-year recurrence-free survival rates were 50%, 35%, 19% and 19%, respectively. Among patients with localised intermediate-grade disease survival for those with gastric NHL was better than for those with intestinal lymphoma. Because it is population-based, our study cohort was not subjected to exclusion due to age, performance scale, etc. and therefore provides a more realistic picture of the occurrence and presentation of as well as prognosis for lymphoma in the population.


Br. J. Cancer (1989), 60, 745–750 © The Macmillan Press Ltd., 1989

### Primary gastrointestinal non-Hodgkin's lymphoma in a population-based registry

R. Otter[1], R. Bieger[2], Ph. M. Kluin[3], J. Hermans[4] & R. Willemze[5] on behalf of the Study Group

[1]*Comphrensive Cancer Centre West, Schipholweg 5[A], 2316 XB Leiden, the Netherlands;* [2]*Department of Internal Medicine, Bronovo Hospital, Bronovolaan 5, 2597 AX Den Haag, the Netherlands;* [3]*Laboratory of Pathology, State University Leiden, Wassenaarssweg 62, 2333 AL Leiden, the Netherlands;* [4]*Department of Medical Statistics, State University Leiden, Postbus 9512, 2300 RA Leiden, the Netherlands; and* [5]*Department of Haematology, University Hospital, Postbus 9600, 2300 RC Leiden, the Netherlands.*

**Summary** In a population-based registry of 580 patients with non-Hodgkin's lymphoma (NHL) 54 patients had a primary gastric lymphoma, 42 an intestinal, 113 a primary extranodal lymphoma localised elsewhere than in the gastrointestinal tract and 371 a primary nodal NHL. Histological specimens were reviewed by a panel of pathologists and classified according to the Kiel classification and the International Working Formulation. The 4-year survival rates for primary gastric, intestinal, other extranodal and nodal NHL ranged from 50 to 60%; the 4-year recurrence-free survival rates were 50%, 35%, 19% and 19%, respectively. Among patients with localised intermediate-grade disease survival for those with gastric NHL was better than for those with intestinal lymphoma. Because it is population-based, our study cohort was not subjected to exclusion due to age, performance scale, etc. and therefore provides a more realistic picture of the occurrence and presentation of as well as prognosis for lymphoma in the population.

In the Netherlands an estimated 1000 new cases of non-Hodgkin's lymphoma (NHL) are diagnosed every year (SOOZ, 1985), 400 of which are primary extranodal lymphomas (Haak *et al.*, 1986) while 100–200 are primary gastrointestinal NHLs (Brady & Asbell, 1980; Freeman *et al.*, 1972; Haber & Mayer, 1988).

Most previous data on primary gastrointestinal lymphoma were obtained from retrospective studies based on patient records in a given hospital over a period of many decades (Brooks & Enterline, 1983; Economopoulos *et al.*, 1985; Haber & Mayer, 1988; Shiu *et al.*, 1986; Thorling, 1984). A variety of histological classification systems led to controversial conclusions with regard to the association between histological type and prognosis (Dworkin *et al.*, 1982; Weingrad *et al.*, 1982). In contrast to these hospital studies we analysed the clinical and histological presentation as well as the survival data recorded in a population-based registry. The advantage of such a registry is that both bias due to hospital referral policy and selection of patients are avoided. In the Netherlands, eight comprehensive Cancer Centres (CCC) cover the entire country geographically. In 1981, the regional NHL Study Group of CCC West (1.58 million inhabitants) started a population-based registry, as defined by MacCennan *et al.* (1978), of all new NHL cases. The primary gastric and intestinal lymphomas in the registry were investigated and compared with two other categories: other primary extranodal lymphomas and nodal lymphomas. The present paper reports the results of this analysis of the four categories of lymphoma in the population-based registry with special attention directed to gastric and intestinal NHLs.

### Materials and methods

A population-based registry of all new cases of NHL within the region of the CCCW was started on 1 June 1981, and is still ongoing. However, for this study, data entry of new cases ended on 1 September 1986, while entry of follow-up data ended on 31 December 1987. All hospitals (15) and pathological laboratories (9) in the region participated through the NHL Study Group, which consists of 45 specialists. Collection of data and quality controls were performed by the NHL registrars of the CCCW. Included were all newly diagnosed patients with NHL except those with mycosis fungoides, acute lymphoblastic leukaemia, classical chronic lymphocytic leukaemia and multiple myeloma and those first diagnosed post-mortem.

In this study the data were divided into four groups on the basis of the primary involved site coded according to the International Classification of Diseases for Oncology (ICD-O, 1976). A distinction was made between primary extranodal lymphomas and primary nodal NHL; primary extranodal NHL was further subdivided into primary gastric, intestinal and other primary extranodal lymphoma (primary gastrointestinal NHL excluded). The NHL was considered a primary extranodal lymphoma when at initial clinical examination the patient's symptoms were caused mainly by extranodal NHL involvement. When an organ localisation was diagnosed during staging procedures or later in the course of the disease, the lymphoma was not subsequently considered a *primary* extranodal lymphoma. Patients with primary gastrointestinal lymphoma were defined according to Lewin *et al.* (1978). The initial diagnosis and histological classification of all cases were made by local pathologists. A panel of four pathologists reviewed all newly diagnosed cases weekly. All NHLs were registered, but the tumours were only classified if material was available for additional immunological and enzyme-histochemical studies (Haak *et al.*, 1986). In most other cases, in particular large cell tumours, the character of the lymphoma was confirmed by immunostaining for $CD_{45}$-leukocyte common antigen on paraffin slides (Warnke *et al.*, 1983). The NHLs were classified according to the Kiel system (Lennert, 1978) with some modifications for intermediate lymphocytic lymphoma and true histiocytic lymphoma (Haak *et al.*, 1986) which were considered additional entities. Lymphomas were graded as low, intermediate and high-grade malignancy according to the International Working Formulation (National Cancer Institute, 1982). Intermediate lymphocytic lymphoma was considered as intermediate-grade, true histiocytic lymphoma as high-grade malignancy.

All NHL patients were staged according to the Ann Arbor Classification system (Carbone *et al.*, 1971); staging was considered adequate only when physical examination (Waldeyer's ring included), surgical and/or endoscopy reports and additional examinations such as bone marrow biopsy, X-rays (at least chest X-ray), CT-scan of the abdomen or lymphangiography and isotope scans of the liver and spleen were performed. All stage $II_E$ primary gastrointestinal lymphomas were further subdivided according to Musshoff (1977) into stages $II_{1E}$ and $II_{2E}$. We considered gastric NHL

Correspondence: R. Otter.
Received 22 November 1988; and in revised form 6 March 1989.

with direct extension into adjacent organs (pancreas, liver) as stage $IV_E$ disease, in accordance with the findings of Weingrad *et al.* (1982).

Although the NHL Study Group reached a general consensus on various treatment modalities at the start of the registry (Table I), no special protocol for the type of surgery for patients with primary gastrointestinal lymphoma was followed. Usually a partial gastrectomy was performed when the lymphoma was localised in the stomach. Surgical procedures for intestinal localisations were total resection of the involved site when cure was considered possible or partial resection. Lymphoblastic lymphomas were treated as acute lymphoblastic leukaemia with an induction, consolidation and maintenance drug scheme. Response to treatment was assessed according to standard criteria.

Survival time and recurrence-free survival time were calculated from the date of diagnosis. Death with tumour was considered the endpoint for survival and recurrence of disease or death the endpoint for recurrence-free survival. Comparison of survival curves was performed with the log rank (Matthews & Farewell, 1985). Comparison of survival rates at specific time points was done using Greenwood's equation for the standard error of the survival rate. Contingency tables were analysed with the $\chi^2$ test.

### Results

Out of 640 new cases of NHL registered from 1 June 1981, to 1 September 1986, 580 were evaluable; the registration forms of 60 patients were incomplete, 30 of whom were lost to follow-up.

### *Gastric and intestinal* vs *other extranodal* vs *nodal NHL*

Table II lists some patients' characteristics for the two main groups: primary extranodal and primary nodal lymphoma. The extranodal group is further subdivided into primary gastric, intestinal and other extranodal NHLs. Among the 580 evaluable patients, 209 had primary extranodal lymphoma, 54 of which were primary gastric and 42 primary intestinal lymphoma. Thirteen of the intestinal NHLs were localised in the small intestine, 16 in the large bowel and rectum, three at multiple intestinal sites and 10 in the mesentery. The mesenterial localisations (10 patients) were considered to be primary intestinal lymphomas in accordance with the literature (Dragosics *et al.*, 1985; Haber & Mayer, 1988; Weingrad *et al.*, 1982). Because the numbers of patients per subsite were too small, we analysed these patients with intestinal NHL as one group, although it is a very heterogeneous group.

*Sex and age* Sex distribution differed between the four groups ($\chi^2 = 12.9$; d.f. = 3; $P = 0.05$). Adjusted for age and sex for the regional population there was no difference in sex distribution within a group. Although the median ages for the four groups were similar, 10 patients of the 42 patients with primary intestinal NHL were younger than 20 years of age.

*Histology* The histological subtypes are summarised in Table III. Twenty-four per cent of all NHLs were not classified due to the lack of frozen material, in particular those diagnosed on the basis of endoscopic biopsy specimens; histological diagnosis was established on endoscopic biopsy alone in 21 out of 54 gastric lymphoma and four out of 42 intestinal lymphoma. Among the classified lymphomas, high-grade malignancy was encountered more often in primary gastrointestinal lymphomas ($n = 22$ out of the 68, 32%) – in particular the intestinal ($n = 14$ out of the 33, 43%) – than in the group with other extranodal NHL (15%) or primary nodal NHL (13%) ($\chi^2 = 16.9$; d.f. = 4; $P = 0.002$). Taking the Kiel classification into consideration the type distribution of the gastric lymphoma was different compared to the other groups, with a predominance of diffuse centroblastic lymphoma (45%). Half of the mesenterial lymphomas were of the follicular type; this type was very infrequent in the stomach.

*Stage* Stage I disease was found in 30–40% of the cases of extranodal NHL and only 14% of those with nodal lymphoma. In parallel, 68% of the nodal NHLs were stage IV compared to 20 to 50% of the extranodal lymphomas.

The three patients with gastric lymphoma stage $III_E$ had NHL of the cardia and pathological regional lymph nodes just above and below the diaphragm near the cardia. Sixteen patients with gastric lymphoma had stage $IV_E$ disease: 10 because of a positive bone marrow biopsy without NHL involvement other than the stomach and six because of additional solitary extranodal involvement without lymph node involvement. Eight patients with intestinal NHL had stage $IV_E$ disease as indicated by multiple gastrointestinal localisations ($n = 3$), a solitary cervical lymph node ($n = 3$) and a positive bone marrow biopsy without lymph node involvement ($n = 2$).

Adequate staging procedures as defined by the NHL Study Group were performed in an equal percentage of the cases of primary gastric, intestinal lymphoma and nodal NHL (74%, 67% and 69%, respectively; Table II) and in 59% of the cases of other primary extranodal lymphomas.

*Survival and recurrence-free survival* The survival and recurrence-free survival times for patients with gastric and intestinal lymphoma in relation to other extranodal and nodal NHL are shown in Figure 1. No important differences in survival were present between the four groups. Up to 42 months the intestinal group had the poorest survival. The survival rate at 48 months, however, was similar for the four groups. The recurrence-free survival results were markedly influenced by the large number of patients who never achieved complete remission. At 48 months the gastric lymphoma group, in particular, had a better prognosis compared to patients with other extranodal or nodal NHL (Greenwood's equation; $P < 0.05$; Figure 1b). In the next section, our analysis of gastric and intestinal lymphomas is presented.

**Table I** Radiotherapeutic and chemotherapeutic protocols for NHL patients, listed according to stage and grade of malignancy

| | *Working formulation* | | |
|---|---|---|---|
| *Stage* | *Low* | *Intermediate* | *High* |
| I | wait and see radiotherapy (35–40 Gy)/ chlorambucil | radiotherapy (35–40 Gy) | CHOP |
| $II_1$ | see stage I | CHOP | CHOP |
| $II_2$–IV | CVP | CHOP | CHOP |

CVP: cyclophosphamide 300 mg $m^{-2}$ orally on days 1–5, vincristine 1.4 mg $m^{-2}$ (max. 2 mg) i.v. on day 1, prednisone 60 mg orally on days 1–5. CHOP: cyclophosphamide 750 mg $m^{-2}$ i.v. on day 1, adriamycine 50 mg $m^{-2}$ i.v. on day 1, vincristine 1.4 mg $m^{-2}$ (max. 2 mg) i.v. on day 1, prednisone 60 mg orally on days 1–5.

**Table II** Characteristics of patients with NHL ($n = 580$)

| | *Primary extranodal NHL* | | | *Nodal NHL* |
|---|---|---|---|---|
| *Characteristics* | *Stomach* | *Intestine* | *Other* | |
| Number of patients | 54 | 42 | 113 | 371 |
| Sex: M/F | 33/21 | 22/20 | 39/74 | 186/185 |
| Age: | | | | |
| Median in years | 65 | 65 | 66 | 64 |
| Range | 20–87 | 0–95 | 4–94 | 0–91 |
| Stage* | | | | |
| I | 21 (15) | 12 ( 7) | 35 (17) | 51 ( 34) |
| II | | | 20 (16) | 67 ( 53) |
| $II_1$ | 6 ( 4) | 10 ( 8) | | |
| $II_2$ | 8 ( 7) | 12 ( 8) | | |
| III | 3 ( 3) | 0 ( 0) | 1 ( 1) | 82 ( 52) |
| IV | 16 (11) | 8 ( 5) | 57 (33) | 171 (118) |
| Total | 54 (40) | 42 (28) | 113 (67) | 371 (257) |

*The number of patients adequately staged is given in parentheses.

**Table III** Histological classification

| | Primary extranodal NHL | | | Nodal NHL |
|---|---|---|---|---|
| | Primary gastrointestinal NHL | | Other | |
| | Stomach | Intestine | | |
| *Classified* | **35 (100%)** | **33 (100%)** | **73 (100%)** | **300 (100%)** |
| Low grade | 2 ( 5%) | 9 ( 27%) | 20 ( 27%) | 93 ( 31%) |
| Lymphocytic | – | – | – | 10 |
| Lymphoplasmocytoid immunocytoma | 1 | 1 | 11 | 15 |
| Follicular centroblastic/centrocytic | 1 | 8 | 9 | 68 |
| Intermediate grade | 24 ( 69%) | 9 ( 27%) | 39 ( 54%) | 161 ( 54%) |
| Diffuse centroblastic/centrocytic | 5 | 1 | 6 | 23 |
| Centrocytic | 1 | – | 2 | 4 |
| Intermediate lymphocytic | 1 | – | 1 | 15 |
| Follicular centroblastic | – | – | 1 | 13 |
| Diffuse centroblastic | 16 | 4 | 23 | 92 |
| Immunocytoma pleomorphic | 1 | 4 | 6 | 14 |
| High grade | 8 ( 23%) | 14 ( 43%) | 11 ( 15%) | 40 ( 13%) |
| Immunoblastic | 6 | 2 | 8 | 17 |
| Lymphoblastic | – | 3 | – | 13 |
| Burkitt's | 1 | 6 | – | 2 |
| True histiocytic | 1 | 3 | 3 | 8 |
| Others | 1 ( 3%) | 1 ( 3%) | 3 ( 4%) | 6 ( 2%) |
| Lennert's lymphoma | – | – | 2 | 6 |
| T-medium sized lymphoma | 1 | 1 | 1 | – |
| *Non-classified* | **19** | **9** | **40** | **71** |
| *Total* | **54** | **42** | **113** | **371** |

**Figure 1** Survival (**a**) and recurrence-free survival (**b**) rates for patients with primary gastric, intestinal, extranodal lymphoma other than gastrointestinal lymphoma and nodal NHL (the number of patients at risk is given in parentheses).

*Gastric* vs *intestinal NHL, prognostic factors*

*Treatment* The treatment modalities are listed in Table IV. The majority of the patients with gastric NHL (61%) and nearly all patients with intestinal lymphoma (90%) underwent surgery. In our study, patients with primary gastric NHL stage $I_E$ or stage $II_{1E}$ only achieved complete remission after surgery.

*Grade of malignancy (according to the Working Formulation)* Too few patients had low-grade gastric lymphoma (Table III). For this reason wer focussed on intermediate-grade and high-grade NHL. Although the survival rates for patients with intermediate-grade gastric NHL and intermediate-grade intestinal lymphoma did not differ significantly within the first 2 years of follow-up (log rank = 2.41; d.f. = 1; $P = 0.12$) they did at 4 years (Greenwood; $P < 0.05$). There was no difference in survival between high-grade gastric and intestinal lymphoma. (See Figure 2.)

*Stage* The survival rates for stage $I_E$ and $II_{1E}$ patients with gastric NHL were similar; however, survival for stage $II_{2E}$ patients was as poor as that for stage $IV_E$ disease. For this reason, we compared the survival data for patients with localised lymphoma (stage $I_E$ + stage $II_{1E}$) with those with more extensive disease (stages $II_{2E}$, $III_E$ and $IV_E$). Localised gastric lymphoma tended to have a better survival rate than localised intestinal NHL although the difference was not significant (log-rank = 1.74; d.f. = 1; $P = 0.18$). Survival rates for patients with extensive disease were equal for those with gastric and intestinal NHL. (See Figure 3.)

Figures 2 and 3 suggest that there might be a difference in survival between localised intermediate-grade gastric and intestinal lymphoma; despite the small numbers of patients ($n = 14$ and $n = 5$), Figure 4 shows a significant difference (log rank = 4.41; d.f. = 1; $P = 0.03$).

### Discussion

In this study we analysed all primary gastrointestinal lymphomas diagnosed in a clearly defined population over a short period of time. Since most studies are based on a hospital registry or clinical investigations instead of a population-based registry, our patients represent a wider age range (0–94 years) than is found in the reports of others (Dragosics *et al.*, 1985; Economopoulos *et al.*, 1985). Moreover, since a

**Table IV** Treatment modalities related to the stage of the primary gastrointestinal NHL

| | Surgery | | No surgery | |
|---|---|---|---|---|
| | $I_E + II_{IE}$ | $II_{2E} + III_E + IV_E$ | $I_E + II_{IE}$ | $II_{2E} + III_E + IV_E$ |
| Stomach | | | | |
| no additional treatment | 8 ( 8) | 1 ( 0) | – | 1 (0) |
| chemotherapy alone | 7 ( 5) | 6 ( 4) | 2 (0) | 15 (1) |
| radiotherapy alone | 6 ( 6) | – | 3 (2) | 0 (0) |
| radiotherapy + chemotherapy | 1 ( 1) | 4 ( 0) | – | – |
| Total | 22 (20) | 11 ( 4) | 5 (2) | 16 (1) |
| Intestine | | | | |
| no additional treatment | 5 ( 1) | 4 ( 0) | – | – |
| chemotherapy alone | 16 ( 7) | 9 ( 4) | – | 3 (1) |
| radiotherapy alone | 1 ( 1) | – | – | – |
| radiotherapy + chemotherapy | | 3 ( 3) | – | 1 (0) |
| Total | 22 ( 9) | 16 ( 7) | – | 4 (1) |

The number of patients who achieved complete remission is given in parentheses.

**Figure 2** Survival of patients with primary gastric ($n = 54$) and primary intestinal NHL ($n = 42$) subdivided into those wtih intermediate-grade and those with high grade malignancy according to the Working Formulation (the number of patients at risk is given in parentheses).

**Figure 3** Survival of patients with primary gastric ($n = 54$) _nd primary intestinal NHL ($n = 42$) subdivided into those wtih localised and those with more disseminated disease (the number of patients at risk is given in parentheses).

**Figure 4** Survival of patients with localised intermediate-grade NHL of the stomach ($n = 14$) and the intestine ($n = 5$).

population-based registry can be studied after a shorter period of registration diagnostic procedures and therapeutic approach are less likely to vary within the study period.

In the majority of series published (Green et al., 1979; Haber & Mayer, 1988; Weingrad et al., 1982), primary gastrointestinal lymphoma exhibits a marked predilection for males. In our registry and other reports (Brady & Asbell, 1980; Dragosics et al., 1985) both sexes were equally represented. The incidence of intestinal NHL varies considerably (Aozasa et al., 1988; Haber & Mayer, 1988; Weingrad et al., 1982), particularly the relative incidence for the small intestine: from 9% (Dragosics et al., 1985) to 59% (Weingrad et al., 1982) of all primary gastrointestinal lymphomas. These differences might be explained by selection of patients: some studies did not include children (Dragosics et al., 1985; Economopoulos et al., 1985; Weingrad et al., 1982), whereas Burkitt's and lymphoblastic lymphomas are relatively more frequent in young people (Haber & Mayer, 1988).

Grading according to the International Working Formulation appeared to be related to survival in our series (Figures 2 and 4). In other studies only a weak (or no) association was found between prognosis and histological type as given by the Rappaport and/or Lukes-Collins classification system (Dragosics et al., 1985; Dworkin et al., 1982; Haber & Mayer, 1988; Thorling, 1984). When the different classification systems were applied to the same histological material, the Kiel classification system and the International Working Formulation were found to have the better discriminating capabilities (Dragosics *et al.*, 1985; Dworkin *et al.*, 1982).

We found a better 1 year survival rate for nodal NHL but a higher 4-year survival rate for patients with primary gastric lymphoma (Figure 1a). Probably, the initially better but later poorer survival of patients with nodal NHL is caused by the higher percentage of low-grade malignancies among patients with nodal NHL compared to those with primary gastric lymphoma (Table III). It is well known that although patients with low-grade NHL can achieve CR by means of therapy, relapse and death with tumour can rarely be avoided (Ciampi *et al.*, 1981).

Our stage distribution for primary gastrointestinal lymphoma is in accordance with observations of some authors (Blackledge *et al.*, 1979; Haber & Mayer, 1988). Notably higher percentages for stage $I_E$ and $II_E$ disease will be found when the definition of primary gastrointestinal lymphoma is based on the more rigid criteria proposed by Dawson *et al.* (1961), who excluded cases with bone marrow localisation (Dragosics *et al.*, 1985). In our study, 10 out of 16 patients with stage IV gastric NHL and two out of eight with stage IV intestinal NHL would have had stage $I_E$ disease according to physical examination and CT-scan of the abdomen but became stage IV after bone marrow biopsy. Because of the lack of nodal involvement we believe that these patients should nevertheless be included in the primary gastrointestinal lymphoma group. Once more these data emphasise the importance of adequate staging procedures (Come & Chabner, 1979).

Since the Ann Arbor system was not considered suitable for the staging of gastrointestinal lymphomas, other staging systems were set up (Blackledge *et al.*, 1979; Crowther & Rankin, 1983; Musshoff, 1977). In our study the modification of the Ann Arbor system by Musshoff (1977) proved to be useful as a prognostic determinant, which is in accordance with the findings of others (Dawson *et al.*, 1961; Weingrad *et al.*, 1982). Analysis of the relation between stage and prognosis showed that survival was better for patients with localised (stages I and $II_{1E}$) primary gastric lymphoma than for those with extensive disease. Patients with localised gastric lymphoma tended to have a better survival than those with intestinal NHL.

The best treatment for primary gastric lymphoma is a matter of active debate. Many investigators (Dworkin *et al.*, 1982; Gospodarowicz *et al.*, 1983; Haber & Mayer, 1988; Shiu *et al.*, 1986) claim that surgery plus postoperative radiotherapy gives the best chance of survival for those with limited disease. However, in these series, irradiation was consistently given postoperatively, so it is impossible to estimate its impact on survival. More recently, chemotherapy alone or in combination with surgery and/or radiotherapy has been used for limited as well as more extensive disease (Brooks & Enterline, 1983; Economopoulos *et al.*, 1985; Gospodarowicz *et al.*, 1983; Haber & Mayer, 1988; Maor *et al.*, 1984; Weingrad *et al.*, 1982). In our series chemotherapy alone did not seem to be successful in achieving CR (11%), in contrast to the combination surgery plus chemotherapy with or without radiotherapy (52%). The choice of surgery as treatment modality was apparently not based on the stage of disease (Table IV). Complications such as perforation and gastrointestinal bleeding have been described (List *et al.*, 1988; Sheridan *et al.*, 1985; Weingrad *et al.*, 1982) after chemotherapy. None of our patients receiving chemotherapy developed such complications, nor did those described by Economopoulos (Economopoulos *et al.*, 1985). The possible beneficial effect of curative or debulking surgery before chemotherapy, i.e. to prevent perforation and gastrointestinal bleeding, has not yet been established.

Unlike nodal lymphomas, many primary gastric and intestinal lymphomas are presumed to behave clinically as unifocal tumours presenting in a localised fashion which might, therefore, be curable by local radical treatment (Brooks & Enterline, 1983; Dragosics *et al.*, 1985; Haber & Mayer, 1988). However, the far greater likelihood of systemic rather than isolated recurrence in patients with gastric and intestinal NHL (Haber & Mayer, 1988; Figures 1b, 3 and 4) raises reservations about the lack of dissemination at presentation and therefore the justification for adjuvant local irradiation. Optimal management of patients with primary gastric and intestinal lymphoma can be determined only by multicentre prospective trials.

The authors thank Mrs J. Stuart for data collection, Mrs A.W. Lyklema for the statistical analyses, Mrs N.J. Muller for preparing the manuscript and Mrs G. Bieger-Smith for correcting the English text.

The Study Group consisted of the following members. University Hospital of Leiden: R.J.L. Caspers, A. Chin, J.C. Kluin-Nelemans, J.W.H. Leer, E.M. Noordijk, A. Snijders-Keilholz, E.M. v.d. Steen, L.J. Verboom-van Tienen. Sint Antoniushove: J. Blankespoor, M.G. Herben, F. Steenwinkel. Bethlehem Hospital: A. Folmer. Bleuland Hospital: K.J. Heering, J.F. Keuning, J.B. Rahder. Bronovo Hospital: M. Voortman. Sint Elisabeth Hospital: J.J. Calame, W.G. Peters, P.C.M. Rosekrans. Diaconessenhuis Leiden: M.C.B. Gorsira, H. van Slooten. Diaconessenhuis Voorburg: J.R. v.d. Mey. Sint Jozeph Hospital: R.F.A. Simonis. Municipal Hospital Leyenburg: W.B.J. Gerrits, K. van Groningen, H. Haak, B.A. Kazzaz, H. Kerkhofs, J.A.L. Metsaars Reinier de Graaf Gasthuis: A. Houwing, A.H. Mulder, J. de Regt. Rode Kruis Hospital: W.A. van Deijk, G.E. de Greef, P.J. Spaander. SSDZ: S.H. Overdiep, M.M. Van de Sandt, M.L. Spreutels. Westeinde Hospital: A.P.R. Blok, G. Booij, E.C.M. Ooms, R.W. Veldhuizen, R. Vriesendorp.

### References

AOZASA, K., UEDA, T., KURATA, A. & 6 others (1988). Prognostic value of histology and clinical factors in 56 patients with gastrointestinal lymphomas. *Cancer*, **61**, 309.

BLACKLEDGE, G., DODGE, O. & CROWTHER, D.(1979). A study of gastrointestinal lymphoma. *Clin. Oncol.*, **5**, 209.

BRADY, W. & ASBELL, O. (1980). Malignant lymphoma of the gastrointestinal tract. *Radiology*, **137**, 291.

BROOKS, J. & ENTERLINE, H. (1983). Primary gastric lymphomas: a clinico-pathologic study of 58 cases with long term follow-up and literature review. *Cancer*, **51**, 707.

CARBONE, P., KAPLAN, H., MUSSHOFF, K., SMITHERS, D. & TUBIANA, M. (1971). Report of the committee on Hodgkin's disease staging classification. *Cancer Res.*, **31**, 1860.

CIAMPI, A., BUSH, R.S., GOSPODAROWICZ, M. *et al.* (1981). An approach to classifying prognostic factors related to survival experience for non-Hodgkin's lymphoma patients; based on a series of 982 patients, 1967–1975. *Cancer*, **305**, 735.

COME, S. & CHABNER, B. (1979). Staging in non-Hodgkin's lymphoma: approach, results and relationship to histopathology. *Clin. Haematol.*, 645.

CROWTHER, D. & RANKIN, E.M. (1983). Staging patients with non-Hodgkin's lymphoma. *Br. J. Haematol.*, **52**, 357.

DAWSON, I., CONNER, J. & MORSON, B. (1961). Primary malignant lymphoid tumors of the intestinal tract: report of 37 cases with a study of factors influencing prognosis. *Br. J. Surg.*, **49**, 80.

DRAGOSICS, B., BAUER, P. & RADASZKIEWICZ, T. (1985). Primary gastrointestinal non-Hodgkin's lymphomas. A retrospective clinicopathologic study of 150 cases. *Cancer*, **55**, 1060.

DWORKIN, B., LIGHTDALE, S., WEINGRAD, D. & 5 others (1982). Primary gastric lymphoma. *Dig. Dis. Sci.*, **11**, 986.

ECONOMOPOULOS, T., ALEXOPOULOS, C., STATHAKIS, N. & 4 others (1985). Primary gastric lymphoma. The experience of a general hospital. *Br. J. Cancer*, **52**, 391.

FREEMAN, C., BERG, J. & CUKLER, S. (1972). Occurence and prognosis of extranodal lymphomas. *Cancer*, **29**, 252.

GOSPODAROWICZ, M.K., BUSH, R.S., BROWN, T.C. & CHUA, T. (1983). Curability of gastrointestinal lymphoma with combined surgery and radiation. *Int. J. Radiat. Oncol. Biol. Phys.*, **9**, 3.

GREEN, J., DAWSON, A., JONES, P. & BRUNT, P. (1979). The presentation of gastrointestinal lymphoma: study of a population. *Br. J. Surg.*, **66**, 798.

HAAK, H.L., KLUIN, P.M., MEYER, C.J.L.M. & 10 others (1986). Population-based registration of non-Hodgkin lymphoma in the region covered by the Comprehensive Cancer Centre West. *Neth. J. Med.*, **29**, 105.

HABER, D.A. & MAYER, R.J. (1988). Primary gastrointestinal lymphoma. *Semin. Oncol.*, **15**, 154.

ICD-O (1976). *International Classification of Diseases for Oncology*. WHO: Geneva.

LENNERT, K. (1978). *Malignant Lymphomas Other than Hodgkin's Disease*. Springer-Verlag: Berlin.

LEWIN, K., RANCHOD, M. & DORFMAN, R. (1978). Lymphoma of the gastrointestinal tract. A study of 117 cases presenting with gastrointestinal disease. *Cancer*, **42**, 693.

LIST, A.F, GREER, J.P., COUSAR, J.C. & 6 others (1988). Non-Hodgkin's lymphoma of the gastrointestinal tract: an analysis of clinical and pathological features affecting outcome. *J. Clin. Oncol.*, **6**, 1125.

MACCENNAN, R., MUIR, C.S., STEINITZ, R., WINKLER, A. & DAVIS, W. (1978). Purpose of cancer registration. In *Cancer Registration and Its Techniques*, MacCennan, R. Davis, W. (eds) p. 7. IARC: Lyon.

MAOR, M., MADDUX, B., OSBORNE, B. & 7 others (1984). Stage $I_E$ and $II_E$ non-Hodgkin's lymphomas of the stomach: comparison of treatment modalities. *Cancer*, **54**, 2330.

MATTHEWS, D.E. & FAREWELL, V. (1985). *Using and Understanding Medical Statistics*. Karger: Basel.

MUSSHOFF, K. (1977). Klinische Stadieneinteilung der Nicht-Hodgkin-Lymphome. *Strahlentherapie*, **153**, 218.

NATIONAL CANCER INSTITUTE SPONSORED STUDY OF CLASSIFICATION OF NON-HODGKIN'S LYMPHOMAS (1982). Summary and description of a working formulation for clinical usage. *Cancer*, **49**, 2112.

SHERIDAN, W., MEDLEY, G. & BRODIE, G. (1985). Non-Hodgkin's lymphoma of the stomach: A prospective pilot study of surgery plus chemotherapy in early and advanced disease. *J. Clin. Oncol.*, **3**, 495.

SHIU, M., NISCE, L., PINNA, A. & 4 others (1986). Recent results of multimodal therapy of gastric lymphoma. *Cancer*, **58**, 1389.

SOOZ (1985). Cancer incidence in the Netherlands, South Eastern Part, 1978–1982, Eindhoven.

THORLING, K. (1984). Gastric lymphomas, clinical features, treatment and prognosis. *Acta Radiol. Oncol.*, **23**, 193.

WARNKE, R.A., GATTER, K.C., FALINI, B. *et al.* (1983). Diagnosis of human lymphoma with monoclonal anti-leukocyte antibodies. *N. Engl. J. Med.*, **309**, 1275.

WEINGRAD, D., DECOSSE, J., SHERLOCK, P. & 3 others (1982). Primary gastrointestinal lymphoma: a 30-year review. *Cancer*, **49**, 1258.